# Furanoic Lipid F-6, A Novel Anti-Cancer Compound that Kills Cancer Cells by Suppressing Proliferation and Inducing Apoptosis

**DOI:** 10.3390/cancers11070960

**Published:** 2019-07-09

**Authors:** Jassim M. Al-Hassan, Yuan Fang Liu, Meraj A. Khan, Peiying Yang, Rui Guan, Xiao-Yan Wen, Mohammad Afzal, Sosamma Oommen, Bincy M. Paul, Divya Nair, Nades Palaniyar, Cecil Pace-Asciak

**Affiliations:** 1 Department of Biological Sciences, Faculty of Science, Kuwait University, Safat 13060, Kuwait; 2 Program in Translational Medicine, Peter Gilgan Centre for Research and Learning (PGCRL), The Hospital for Sick Children, Toronto, ON M5G 0A4, Canada; 3 Department of Palliative, Rehabilitation and Integrative Medicine, The University of Texas MD Anderson Cancer Center, Houston, TX 77030, USA; 4 Zebrafish Centre for Advanced Drug Discovery & Keenan Research Centre for Biomedical Science, Li Ka Shing Knowledge Institute, St. Michael’s Hospital, Unity Health Toronto, Toronto, ON M5B 1W8, Canada; 5 Departments of Lab Medicine and Pathobiology, and Institute of Medical Sciences, Faculty of Medicine, University of Toronto, Toronto, ON M5G 0A4, Canada; 6 Department of Zoology, CMS College, Kottayam 686001, India; 7 Department of Pharmacology, University of Toronto, Toronto, ON M5S 1A8, Canada

**Keywords:** F-6 (furanoic F-acid), Gulf catfish lipids, cancer cell lines, cell proliferation, apoptosis, cell recovery

## Abstract

Identifying novel anti-cancer drugs is important for devising better cancer treatment options. In a series of studies designed to identify novel therapeutic compounds, we recently showed that a C-20 fatty acid (12,15-epoxy-13,14-dimethyleicosa-12,14-dienoic acid, a furanoic acid or F-6) present in the lipid fraction of the secretions of the Arabian Gulf catfish skin (*Arius bilineatus Val.*; AGCS) robustly induces neutrophil extracellular trap formation. Here, we demonstrate that a lipid mix (Ft-3) extracted from AGCS and F-6, a component of Ft-3, dose dependently kill two cancer cell lines (leukemic K-562 and breast MDA MB-231). Pure F-6 is approximately 3.5 to 16 times more effective than Ft-3 in killing these cancer cells, respectively. Multiplex assays and network analyses show that F-6 promotes the activation of MAPKs such as Erk, JNK, and p38, and specifically suppresses JNK-mediated c-Jun activation necessary for AP-1-mediated cell survival pathways. In both cell lines, F-6 suppresses PI3K-Akt-mTOR pathway specific proteins, indicating that cell proliferation and Akt-mediated protection of mitochondrial stability are compromised by this treatment. Western blot analyses of cleaved caspase 3 (cCasp3) and poly ADP ribose polymerase (PARP) confirmed that F-6 dose-dependently induced apoptosis in both of these cell lines. In 14-day cell recovery experiments, cells treated with increasing doses of F-6 and Ft-3 fail to recover after subsequent drug washout. In summary, this study demonstrates that C-20 furanoic acid F-6, suppresses cancer cell proliferation and promotes apoptotic cell death in leukemic and breast cancer cells, and prevents cell recovery. Therefore, F-6 is a potential anti-cancer drug candidate.

## 1. Introduction

Naturally occurring products and preparations have played an important role in the development of therapeutic agents (e.g., steroids, carotenoids, vitamins, alkaloids). Classical examples of clinically-relevant drugs developed based on natural molecules include aspirin, penicillin and many cancer drugs [1,2]. Marine animals have been shown to contain anti-cancer activities [3,4], and several marine-based pharmaceuticals (e.g., cytarabine, trabectedin, eribulin mesylate, and vendotin) have been approved for treating various cancers [5,6]. In the context of discovering anti-cancer compounds, scaleless fishes are particularly attractive because they have evolved to rapidly kill the damaged cells at the injured skin and heal the wounds. Therefore, the dermal surface gel secretions of scaleless fishes could contain novel compounds with unique cytotoxicity. We have been studying the therapeutic potential of epidermal gel like preparations of a scaleless marine-dwelling species of catfish (*Arius bilineatus Val.*) from the Arabian Gulf; these skin secretions promote wound healing, especially related to non-healing foot ulcers, in human [7,8]. Our preliminary studies suggest that gel preparations contain multiple therapeutic activities. Therefore, we embarked on a program to identify specific molecules that are responsible for the biological activities of the epidermal gel preparations (lipids and proteins). Several lipid and protein candidates, including growth factors have been identified [8]. We have identified that a specific component of lipid fraction (Ft-3) is a furan-containing C-20 furanoic fatty acid (F-6), which is one of the most bioactive components of this preparation, and potently activates neutrophil extracellular trap formation (NETosis) [9]. F-6 is 12,15-epoxy-13,14-dimethyleicosa-12,14-dienoic acid [10,11], and has anti-inflammatory properties, in vivo [12]. Apoptosis prevents uncontrolled cell proliferation and induces an anti-inflammatory form of cell death. However, the potential of F-6 as apoptosis inducer has been unknown.

Cancer cells use many survival mechanisms to evade cell death [13]. These mechanisms often involve the activation of cell death-related kinase cascades. Typical cell survival/death pathways involve the activation of mitogen activated protein kinases (MAPKs; e.g., ERK, p38 and Jun N-terminal kinase (JNK)), PI3K-Akt-mTOR, and cell death regulatory kinases (e.g., Akt) [14,15]. These kinases activate various other proteins and specific transcription factors to promote cell proliferation (e.g., Activator protein 1, AP-1; c-Jun:c-Fos dimer), Signal transducer and activator of transcription 1 (STAT1), Activating transcription factor 2 (ATF2)) or cell death (e.g., p53) [16,17]. Akt is a key protein that binds BAX and prevents mitochondrial membrane perforation, which leads to cytochrome c release and activation of caspase 7/9. Apoptotic cell death is then characterized by the activation of the executioner protease, caspase 3, which cleaves and activates various other proteins [18]. For example, activated caspase 3 (cleaved; cCasp-3) cleaves poly-ADP ribosyl polymerase (PARP) to inactivate this DNA repair/survival promoting protein [14]. Therefore, reduction in Akt levels will lead to increase in apoptosis. Apoptosis is a preferred form of cell death because it is an anti-inflammatory type of cell death [18]. Therefore, compounds that promote apoptosis are preferred anti-cancer drug candidates.

Most of the cells that undergo apoptosis die. However, cancer cells have the ability to reverse apoptosis (anastasis) and resume proliferation [19]. This unique ability renders the cancer cells to lessen or nullify the effects of certain cancer drugs. Apoptosis recovery often occurs at the early stages of cell death or at a lower concentration of drugs. Typically, when the apoptosis inducing signal is absent after certain period of time, cancer cells recover and proliferate [20]. Compounds that can prevent the ability of the cancer cells to recover from drug-induced apoptosis could become more effective therapeutic candidates.

Here we hypothesized that F-6 would suppress cell proliferation, induce apoptosis and prevent recovery after the induction of cell death in various cancer cells. For the first time, we show that (Ft-3), a lipid fraction of AGCS, possesses the ability to block the proliferation of 2 human cancer cell lines (one leukemic, and one breast cancer cell lines), in vitro. This report further describes that an active anti-cancer compound present in Ft-3 is F-6 (12,15-epoxy-13,14-dimethyleicosa-12,14-dienoic acid), which prevents cancer cell proliferation (e.g., blocking JNK pathway), induces apoptotic cell death (e.g., suppressing Akt pathway) and prevents cell recovery, in a dose-dependent manner. Therefore, this study uncovers F-6 as a potential anti-cancer drug candidate.

## 2. Materials and Methods

### 2.1. Media and Reagents

RPMI 1640 (Roswell Park Memorial Institute) with L-Glutamins and sodium Bicarbonate (Cat#350-000-CL), fetal bovine serum (FBS), antibiotics (penicillin and streptomycin; PS), phosphate-buffered saline (PBS), trypsin-ethylenediamine tetraacetic acid (trypsin-EDTA) and trypan blue were purchased from Wisent Inc. (St. Bruno, QC, Canada). All other chemicals and reagents were obtained from Sigma-Aldrich (Oakville, ON, Canada) unless otherwise stated.

### 2.2. Extraction and Quantification of F-Acids from the Catfish Skin Preparation (AGCS)

F-acids are mostly present as constituents of some lipids and therefore, these lipids have to be saponified and trans-esterified for their detection and quantification. The gel-like AGCS material is freeze dried and extracted with chloroform:methanol:isopropanol (2:1:0.1 v/v). The total lipids thus obtained are trans-esterified with methanolic sodium methoxide at room temperature overnight. 20 mg AGCS total lipids were dissolved in 6 mL of 1 M sodium methoxide (0.5 g sodium methylate in dry methanol). Methanol was evaporated and the mixture was left overnight at room temperature. Ten mL water was added, and the mixture was extracted with diethyl ether. The ether extract was separated, dried with anhydrous sodium sulphate, filtered and evaporated to dryness. The residue was dissolved in hexane and loaded on a silica gel column (Woelm, Signa-Aldrich, Munich, Germany). Fractions were collected by adding 10 mL hexane (10 mL), 30 mL hexane:diethyl ether (7:3, v/v), 30 mL hexane:diethyl ether (1:1, v/v). Furan fatty acids are present in the hexane: diethyl ether (7:3) fraction among other compounds. The composition of the furan fraction (Ft) is carried out by gas chromatography-mass spectrometry (GC/MS) model 7890 (Agilent technologies, Santa Clara, CA) USA) interfaced with an MS detector (Agilent MSD-5975), and an Agilent Chem Station software (Agilent version A.09.03, Agilent, Santa Clara, CA, USA). Separation is made on a VF-5ms capillary column—30 m, 0.25 mm, 0.25 μM—(Agilent J&W VF-5ms, Agilent Technologies, Santa Clara, CA, USA). High purity helium is used as a carrier gas with a flow-rate of 1.0 mL/min. The operating conditions of the GC are: Inlet temperature of 250 °C, transfer line 270 °C, injection volume 0.2 μL. GC temperature is programmed from 70 °C, held for 1 min, followed by10 °C/min ramp to 160 °C, hold for 2 min; 5 °C/min ramp to 210 °C, hold for 5 min and 3 °C/min ramp to 250 °C.

### 2.3. Identification of the Factors in Ft-3 

We employed the services of the Analytical Facility for Bioactive Molecules at the Hospital for Sick Children, PGCRL building, to analyze various furan-containing preparations extracted from the AGCS supplied by one of the coauthors (JMA-H). Authentic mass spectra have been published previously [9].

For the bioassay the compounds were dissolved in micro-liter amounts of ethanol then diluted with medium just before use and rapidly mixed (the rapid mixing is very important). The final concentration of ethanol in each well was 0.5%. A control well with this ethanol concentration (vehicle control) was used in each experiment. The results with dose responses demonstrate good solubility. This compound is also soluble in DMSO (dimethylsulfoxide), but in the present study ethanol has been used because of ease of evaporation. 

### 2.4. Cell Lines

Cells were purchased directly from ATCC (Manassas, VA, USA) with certificate of authentication; some of the anti-cancer effects presented herein have been reproduced independently from the two Institutions (The Hospital for the Sick Children (SickKids) and MD Anderson Cancer Center (MDACC), with different batches of cells) MCF-7 and MDA MB-231 were purchased on 20160612 and K562 on 20151216. On receipt, the cells were grown and expanded, divided into aliquots and frozen in separate tubes under liquid nitrogen according to the manufacturer’s protocol. Every 3 months one tube from each cell line was taken and cells were grown and tested microscopically for morphology. All the three cell lines were passaged twice/week. Most of the experiments were carried out between 15–20 passages. Cells were maintained by following ATCC guidelines. (i) MCF7 (ATCC® HTB-22™); Organism: Homo sapiens; Cell Type: epithelial; Tissue: mammary gland, breast; derived from metastatic site: pleural effusion /Disease:adenocarcinoma [21] (ii) MDA-MB-231 (ATCC® HTB-26™); Organism: Homo sapiens; Cell Type: epithelial; Tissue: mammary gland/breast; derived from metastatic site: pleural effusion; Disease: adenocarcinoma [22].

### 2.5. Cell Culture and WST Cell Proliferation Assay

We performed WST, a colorimetric assay to assess the cell proliferation under different treatment conditions. This assay is based on the cleavage of a tetrazolium salt (2-(4-Iodophenyl)-3-(4-nitrophenyl)-5-phenyl-2h-tetrazolium chloride) to form formazan (a colored compound) by mitochondrial dehydrogenases in viable cells. As a result, the assay measures the net metabolic activity of the cells. Human cell lines (leukemia K562, breast MCF-7 and MDA MB-231) were purchased from ATCC ((Manassas, VA, USA)). MCF-7 and MDA MB-231 (1×10^4^ cells/well/100 L) were plated a day before experiment in normal growth medium. The next day, medium was replaced with serum free medium (Gibco#11835-030) to starve the cells for 4 h. Cells were deprived of serum for 4 h before treatment with the lipids to eliminate binding of the compounds to serum proteins. After starvation, K562 cells were then centrifuged, the medium was replaced with fresh IMDM media containing 1% (v/v) FBS and 100 units of PS. Cells (1 × 10^4^ in 100 L) were seeded into each well with conditioned medium for overnight (20 h) in a 96-well plate to which the test compounds (-ve control ethanol, Ft-3 and F-6) were added at the concentrations indicated. A volume of 10 L WST-1, cell proliferation assay reagent (Roche Diagnostics, Indianapolis, IN, USA), was added to each well and incubated for 4 h, and then the wells were read on a plate reader at 450 nm with a reference reading at 650 nm. Reference reading at 650 nm was used for subtracting background readouts.

### 2.6. Measuring Cancer Cell Death by Confocal Imaging

To assess the cytotoxicity, we also imaged the live and dead cells by using LIVE/DEAD® Cell Imaging Kit (ThermoFisher, Waltham, MA, USA, 0245, cat#R37601). The kit contains a cell-permeable dye (FITC-fluorescein isothiocyanate); produces green fluorescence at excitation/emission 488/515 nm) for staining of live cells and a cell-impermeable dye for staining of dead and dying cells (Texas Red; produces red fluorescence at excitation/emission 570/602 nm). MCF-7 and MDA-MB-231 cells (1 × 10^4^ cells in a volume of 100 L per well) were plated at day 1 before starting the experiment in normal growth medium. The next day, medium was removed, serum free RPMI medium was added containing 100 units/mL of PS to starve cells for 3 h. K562 cells were starved for 4 h in a tissue culture flask in serum free RPMI (Gibco#11835-030) with 100 units/mL of PS, the cells were then centrifuged, and the medium was replaced with fresh RPMI (containing 1% (v/v) FBS and 100 units/mL PS) medium. Cells (1 × 10^4^ in 100 L) were seeded into each well with conditioned medium for overnight (~20 h) culturing in 96-well plate. After overnight treatment with the test compound, 100 L of 2× Live/Dead cell assay mix (LIVE/DEAD® Cell Imaging Kit, Molecular Probes, Eugene, OR, USA) was added to the cells directly, and incubated for 15 min at room temperature, then the cells were washed and mounted for confocal imaging. 

### 2.7. Luminex Multiplex Assays 

Two kits containing different apoptosis-related proteins were purchased from Millipore-Sigma (Ontario, Canada): 10-plex # 48-660MAG (for phosphoproteins; HSP27, MEK1, Erk, p38, JNK, MSK1, p53, c-Jun, ATF2, STAT1) and 11-plex #48-612MAG (for specific protein expression; GSK3α, GSK3β, p70S6K, TSC2, IGF1R, IRS1, Akt, mTOR, IR, PTEN, RPS6). The assays were carried out according to the manufacturer’s instruction by the Analytical Facility for Bioactive Molecules at the Hospital for Sick Children PGCRL building. For MCF-7 and MDA MB-231 cells, 70,000 cells/well were seeded in 0.2 mL, and grown overnight at 37 °C in 5% CO_2_ incubator. The next morning, cells were starved in RPMI for 4 h. For K562 cells, on the day of assay, cells were starved for 4 h in RPMI medium (without FBS), then the cells were centrifuged and suspended in fresh medium; cells were counted and 250,000 cells/well were seeded in 0.2 mL. Cells were treated with compound for 2.5 h in RPMI with 1% (v/v) FBS in 0.2 mL volume. After treatment, cells were washed with ice-cold PBS once, 35 μL lysate buffer was added with protease inhibitors into each well and stored at −80 °C until use. Duplicates of each sample were used. The protein concentration was measured using 5 μL lysate diluted with 20 μL PBS; 10 μL of diluted lysate was used with Pierce BCA protein assay kit to determine protein concentration.

Same amount of the total protein has been loaded in each condition. The phosphoprotein data was normalized by total protein amount accordingly (7.5 μg protein plated for these assays). Furthermore, to minimize the variability, the phosphoprotein and total proteins of each detected target. The normalized phosphoprotein data was presented in raw MFI (mean fluorescent intensity) and further fold change increase determined by the ratio of kinases MFI between treatment groups to their respective controls in each cell type. The raw intensity and fold change data using (2 for 10-plex # 48-660MAG and 2-6 for 11-plex #48-612MAG) independent experiments were used for generating heatmaps. Furthermore, the phosphoprotein showing higher (fold difference; up-regulated) or lower (fold difference; down-regulated) values than their controls were used for the construction of networks and pathways with the help of GeneGoMetacore™ software (Thomson Reuters, St Joseph, MI, USA). Simplified models are presented to summarize the key changes induced by F-6.

### 2.8. Western Blotting

Serum-starved cells were treated with or without F-6 for 24 h. Treatment was terminated by washing cells with ice-cold PBS buffer. Cell lysates were prepared in buffer containing 20 mM Tris-HCl (pH 7.4), 150 mM NaCl, 1 mM EDTA, 1 mM EGTA, 1% (v/v) Triton X-100, 2.5 mM sodium pyrophosphate, 1 mM glycerophosphate, 1 mM sodium orthovanadate, 1 mM PMSF, and 1 µM leupeptin on ice for 60 min. The lysates were clarified by centrifugation at 15,000× *g* for 15 min at 4 °C. Lysates were subjected to protein assay and kept at −80 °C. Protein (250 µg) was immunoprecipitated with anti-PARP antibody or anti-caspase-3 antibody coupled to protein A-agarose beads. After washing of the immunocomplexes with lysis buffer, SDS-PAGE (sodium dodecyl sulfate-polyacrylamide gel electrophoresis) sample loading buffer was added, and the mixture was boiled for 5 min. After centrifugation, the supernatant was loaded onto 10–12% SDS-PAGE gel and transferred to the Trans-Blot Nitrocellulose membrane (Bio-Rad, Hercules, CA, USA). Protein bands on the nitrocellulose membranes were checked visually with Ponceau S-staining to assure equivalent protein loading/transfer comparing different samples. Membranes were blocked with non-fat dry milk (5%, w/v) in PBS containing 0.5% (v/v) Tween-20 for 1 h at room temperature and then incubated with 1:1000 dilution of anti-PARP and anti-caspase-3 antibodies overnight at 4 °C; secondary antibody of horseradish peroxidase anti-rabbit or anti-mouse antibody was used at 1:2000 dilution. Bound antibodies were detected using enhanced chemilluminescence (ECL) kit and the membranes were exposed to Hyperfilm for ECL. The developed images were further scanned for densitometry analysis by ImageJ software (version 146, NIH, Bethesda, MD, USA).

### 2.9. Cell Recovery Assays

Cells were prepared as described above, and starved for 4 h with serum-free RPMI, then replaced with fresh RPMI with 1% (v/v) FBS (100 units/mL PS) medium. After starving, cells were counted and 10,000 cells/well were seeded into 96-well plates with different dosages of compound in a total volume of 100 μL and incubated in 5% CO_2_ incubator at 37 °C for 3 days with compounds. On the third day, cells were counted in Thermo Fisher Countess II and imaged by Optika Microscope with 20× magnification. After cell counting, these cells were diluted to the least cells observed in the experiment and resuspended in fresh RPMI (with 10% FBS and 100 units/mL PS) medium to grow for next 3 days; the same procedure was repeated every 3 days with cell counting, diluting to the least cell count and resuspension in fresh media up to day 14 of the culture.

### 2.10. Zebrafish Care and General Procedure

Zebrafish strains AB, Tg(mpx:GFP) and Tg(mpeg1:mCherry) are raised and maintained using standard laboratory procedures as described [23]. Embryos are obtained via natural mating and cultured in embryo E2 buffer in 28 ± 0.5 °C incubator. All experiments in this study are conducted according to the ethical guidelines established by the St. Michael’s Hospital Animal Care Committee and Research Ethics Board with approved animal protocol ACC660. 

#### 2.10.1. Compound Treatment

Chemical compounds are dissolved in Ethanol (3 µL) and diluted in 300 µL E2 butter. Fish larvae with tailfin uncut are maintained in 24-well plastic dishes. Each well contains 10 larvae in 700 µL of E2 buffer before addition of compound. Compounds are added to each well right after tailfin transection. Following treatment, neutrophil migration and tailfin regeneration are assessed at certain time points. Non-injected controls are included on every plate.

#### 2.10.2. Tailfin Regeneration 

Fish larvae at 4 days post fertilization (dpf) are anesthetized in E2 buffer containing 0.1 mg/mL Tricaine prior to wounding. Tailfin transection is performed with a 30-gauge needle, sterilized using 70% ethanol prior to use. A single cut is made traversing the entire dorsoventral length of the caudal fin through the end of the notochord. Larvae are incubated for 2 and 6 days at 28 °C. Images are acquired using fluorescence stereomicroscopy (Leica M205 FA). The lengths of tailfin are analyzed using Fiji software (ImageJ, NIH, University of Wisconsin, Madison, WI, USA).

### 2.11. Statistical Analysis

Statistical analysis was performed using GraphPad Prism statistical analysis software (Version 5.0a, San Diego, CA, USA). Data are presented as mean or mean ± standard error of the mean (SEM). Best fit linear regression analysis was performed to represent the data sets; the slopes of the regression lines were compared to zero to identify whether there was a significant relationship existed between Ft-3 or F-6 concentrations and % cancer cell death. For Western blot data, one-sample t-test was applied to compare the intensities of protein bands present in F-6 treatment conditions to their baseline controls. The averaged raw and fold change values of phosphoproteins and specific cell death-related proteins were plotted as heatmaps. The *n*-values and statistical procedures used are mentioned in figure legends. A *p*-value of ≤0.05 was considered to represent statistically significant differences between two conditions.

## 3. Results

### 3.1. A Furan-Containing Ft-3 Lipid Mix Extracted from AGCS Kills Cancer Cells

**Figure 1:**
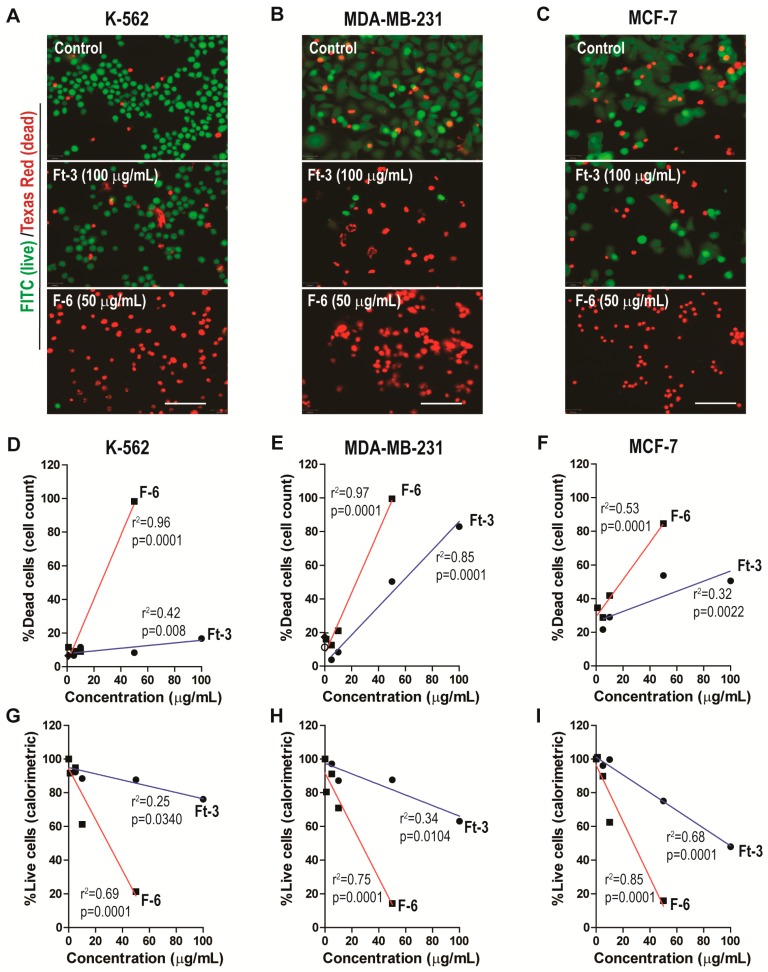
Ft-3 and F-6 dose-dependently kill three cancer cell lines. Leukemic K562, and breast cancer MDA MB-231 and MCF-7 cells were treated with indicated concentrations of Ft-3 and F-6. (**A**–**C**) After 24 hours, death of these cells was analyzed by live (green)/dead (red) fluorescence assay kits. Fluorescence microscopy images show that Ft-3 and F-6 dose-dependently kill cancer cells. Scale bar, 20 μm. (**D**–**F**) Dead cell percentage in each condition was calculated after counting red and green cells. Regression analyses show that both Ft-3 and F-6 dose-dependently kill all three cancer cells. Slope of all the regression lines are greater than 0. The r2 values and *p*-values are indicated on each panel. The *p*-values less than 0.05 are considered to represent statistically significant differences (*n* = 3). (**G**–**I**) Anti-proliferative activity of Ft-3 and F-6 was also analyzed with the WST-1 spectrophotometric detection method. These values confirm the microscopy-based method described in A–F. See supplemental Table S1 and supplementary Figure S2 for Non-linear regression (curve fit) analyses for the estimation of EC50 values.

To determine whether lipid extract (Ft-3) of AGCS kills cancer cells, we incubated 3 human cancer cells (K562, MDA MB-231, and MCF-7) with different concentrations of Ft-3. The live (green)/dead (red) fluorescence imaging (Figure 1A–C) and quantitative data analyses showed that Ft-3 dose-dependently killed both of the breast cancer cell lines (MDA MB-231, at a rate of 0.84%/[μg/mL] with logEC50 = 27.8 μg/mL; r^2^ = 0.98 and MCF-7, 0.3%/[μg/mL] with logEC50 = 23.8 μg/mL; r^2^ = 0.52) more effectively than leukemia cell line (K562, 0.08%/[μg/mL] with logEC50 = 2.9 μg/mL; r^2^ = 0.0.35; all of these slopes are higher than 0, *p* < 0.05; Figure 1D–F and supplementary Figure S2). This dose-dependent effect was apparent from the images and from fluorescence-based cell survival plate reader assays (MDA MB-231, −0.31%/[μg/mL] with logEC50 = 10.4 μg/mL; r^2^ = 0.77, MCF-7, −0.53%/[μg/mL] with logEC50 = 10.1 μg/mL; r^2^ = 0.91 and K562, −0.19%/[μg/mL] with logEC50 = 10.4 μg/mL; r^2^ = 0.76; all the slopes are lower than 0, *p* < 0.05; Figure 1G–I and supplementary Figure S2). Therefore, lipids present in AGCS dose-dependently inhibited proliferation and killed all of these three cell lines, albeit with some differences in their potency for killing specific cell lines (Figure 1; supplemental Table S1 and supplementary Figure S2). 

### 3.2. F-6 (12,15-epoxy-13,14-dimethyleicosa-12,14-dienoic acid) Effectively Kills Cancer Cells

We have previously identified that F-6 present in Ft-3 is a potent inducer of NETosis [9]. To test whether F-6 kills cancer cells, we examined its ability to kill the 3 human cancer cell lines. The live (green)/dead (red) fluorescence imaging (Figure 1A–C) and quantitative data analyses showed that F-6 dose-dependently killed all 3 cancer cell lines (MDA MB-231, 1.82%/[μg/mL] with logEC50 = 49.8 μg/mL; r^2^ = 0.80; MCF-7, 1.10%/[μg/mL] with logEC50 = 10.5 μg/mL; r^2^ = 0.44; and K562, 1.89%/[μg/mL] with logEC50 = 24.7 μg/mL; r^2^ = 0.99 (Figure 1D–F and supplementary Figure S2). At 50 μg/mL concentration, F-6 killed >80% of cells. This dose dependent effect was apparent from the images and from fluorescence-based cell survival plate reader assays (MDA MB-231, −1.56%/[μg/mL] with logEC50 = 31.11 μg/mL; r^2^ = 0.19; MCF-7, −1.67%/[μg/mL] with logEC50 = 50.1 μg/mL; r^2^ = 0.69; and K562, −1.51%/[μg/mL] with logEC50 = 32.2 μg/mL; r^2^ = 0.22; all the slopes are lower than 0, *p* < 0.05; Figure 1G–I and supplementary Figure S2). This result indicated that F-6 is a potent inhibitor of the proliferation of these cancer cell lines. 

Based on the live/dead imaging assays, F-6 was 2.17, 3.67 and 23.92- fold more effective than Ft-3 in killing MDA MB-231, MCF-7, and K562 cancer cells, respectively (calculated from Figure 1D–F and supplementary Figure S2). Based on the colorimetric plate-reader assays, F-6 was 4.98, 3.17, and 8.1-fold more effective than Ft-3 in killing MDA MB-231, MCF-7, and K562 cancer cells, respectively (calculated from supplementary Figure S2 and Figure 1G–I). Therefore, considering both of these assays, F-6 was 3.58, 3.42 and 16.02-fold more effective than Ft-3 in killing MDA MB-231, MCF-7, and K562 cancer cells, respectively. Considering both types of cell death assays, F-6 killed both breast cancer cells (MDA MB-231, 1.69%/[μg/mL with logEC50 = 3.11 μg/mL]; MCF-7, 1.39%/[μg/mL logEC50 = 50.1 μg/mL]) and leukemic cells (K562, 1.70%/[μg/mL] logEC50 = 32.2 μg/mL) to a similar extent. Therefore, F-6 is an effective anti-proliferative compound in cancer cells.

### 3.3. Ft-3 and F-6 Regulate Phosphorylation and Expression of Apoptosis-Related Proteins in K562 Leukemic Cells

Cancer cells survive by differentially regulating various kinase cascades that leads to the expression of different sets of survival or apoptosis regulatory proteins [24].Therefore, to determine whether Ft-3 and F-6 alter specific cell death-related pathways, we incubated cancer cells with these lipids for 2.5 h and examined the phosphorylation of a panel of cell proliferation and apoptosis-related proteins. We also examined the total protein levels of cell death-specific proteins in these lysates by multiplex assays. 

**Figure 2:**
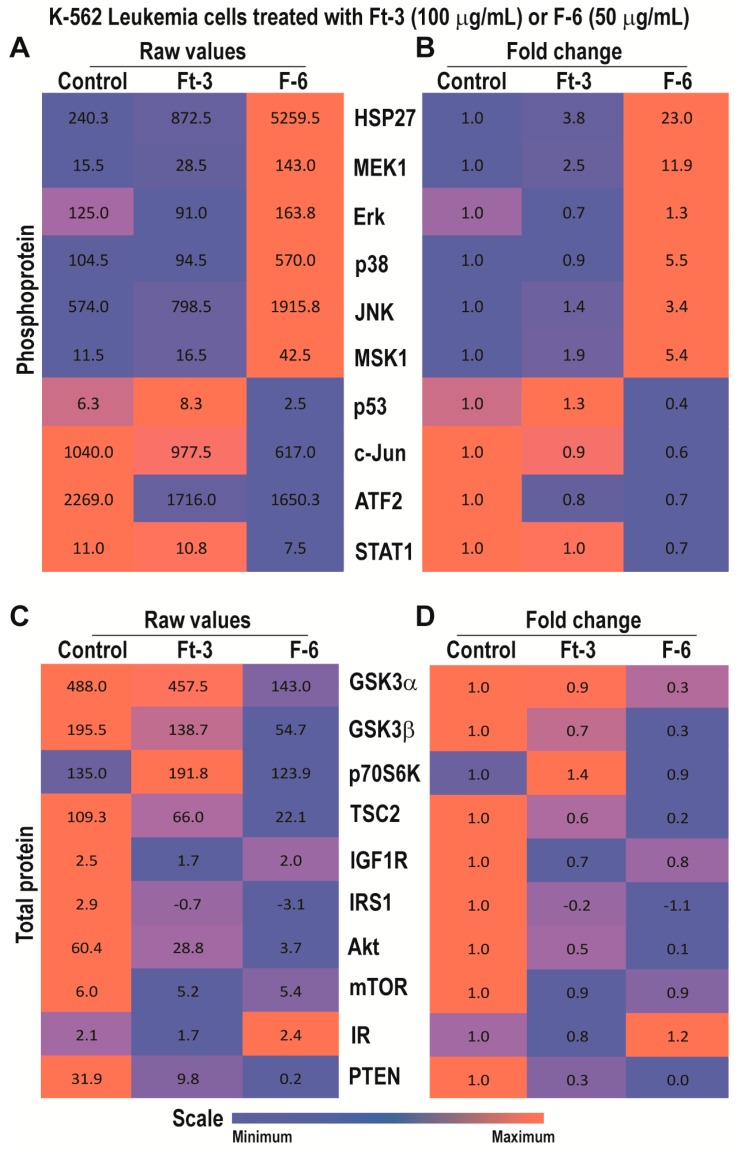
Multiplex Luminex experiments showing the effects of Ft-3 and F-6 on phosphorylated and total apoptotic signaling-related proteins in K562 leukemic cell line. Cells were treated with vehicle control or Ft-3 (100 µg/mL) or F-6 (50 µg/mL) for 2.5 h. (**A**) Levels of phosphorylation of 10 proteins were detected in the lysates of these cells using Luminex assay (*n* = 2). (**B**) Data are also presented as a heat map normalized with vehicle controls and presented as fold differences. (**C**,**D**) Levels of 11 apoptosis-related proteins were detected in the lysates of these cells using Luminex assay (*n* = 3–6). These proteins were standardized to the total protein content of the lysates. (**C**) As of A, but for specific protein levels. (**D**), As of B, but for the fold differences in specific protein levels.

Analyses of phosphoproteins showed that the proteins tested were differentially expressed at baseline; both Ft-3 and F-6 altered the phosphorylation of specific proteins (Figure 2A). Examination of the effect of Ft-3 by fold differences showed that the lipid mixture increased the phosphorylation of MEK1, JNK and MSK1 (Figure 2B). MEK1 and JNK phosphorylate Erk and c-Jun, respectively [25]. However, Ft-3 suppressed these steps (Figure 2B). c-Fos is phosphorylated by Erk or via Erk-MSK1, or p38 depending on the context [26]. Therefore, the suppression of c-Jun phosphorylation by Ft-3 could block the assembly of AP-1 (phosphor forms of c-Fos:c-Jun) to suppress cancer cell proliferation. 

Multiplex assays for total protein levels showed that Ft-3 suppressed several components that induce PI3K-Akt-mTOR pathway (Figure 2C,D; e.g., IR, IRS1, IGF1R, PTEN, Akt, TSC2, mTOR, GSK3; [27]) and new protein translation systems (RPS6; [28]). Ft-3-mediated reduction in Akt levels (Figure 2C,D) can also lead to the induction of apoptosis by intrinsic pathway because Akt is directly involved in preventing apoptosis by binding to BAX [28]. Therefore, these data sets suggest that Ft-3 components primarily interfere with Akt pathway to induce apoptosis.

By contrast, F-6 significantly increased the activation of MEK1 and all three MAPKs (Erk, p38, JNK) and MSK1, but suppressed p53, c-Jun, ATF2, and STAT1 (Figure 2A,B). Downstream targets of Erk and JNK are MSK1/c-Fos and c-Jun, respectively [28]. Phospho c-Jun and c-Fos dimers (AP-1) could activate transcription of cell proliferation related genes [17,29]. Nevertheless, although MSK1 was phosphorylated to higher levels, c-Jun was not phosphorylated during F-6-mediated cell death. These data suggest that F-6 blocks cell proliferation by inhibiting Erk and JNK-mediated AP-1 directed gene activation. The p38 is known to activate HSP27 and CREB-mediated transcription [30]. Hence, F-6 affects Erk and JNK, but not p38-mediated pathway to suppress the proliferation of K562 cells.

Analyses of total protein levels indicated that F-6 suppressed essentially all the components of PI3K-Akt-mTOR pathway and key components of translation machinery (Figure 2C,D). Reduction in Akt can also directly induce apoptosis by allowing BAX to increase mitochondrial membrane permeability and subsequent apoptotic cell death via intrinsic pathway (cytochrome c release, and activation of caspase 7/9, caspase 3). Changes in phosphoprotein and total protein levels suggest that F-6 induces apoptosis in K562 cells via the intrinsic pathway.

### 3.4. Ft-3 and F-6 Regulate Phosphorylation and Expression of Apoptosis-Related Proteins in MDA MB-231 Breast Cancer Cells

**Figure 3:**
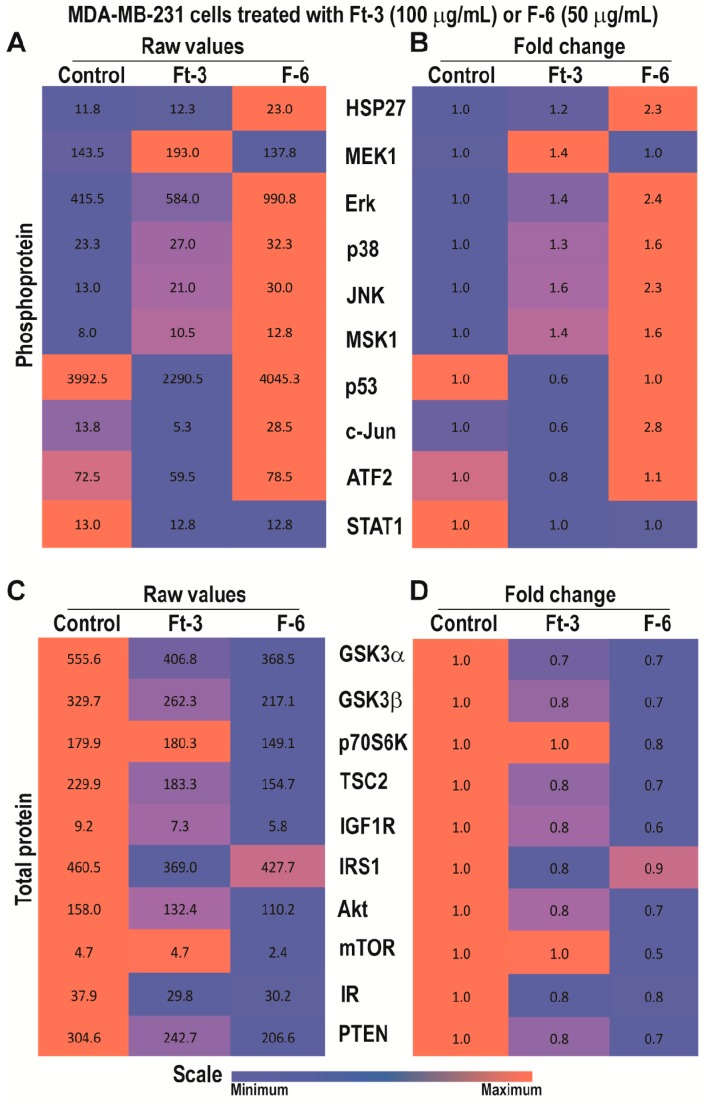
Multiplex Luminex experiments showing the effects of Ft-3 and F-6 on total and phosphorylated signaling-related proteins in MDA MB-231 breast cancer cell line. Cells were treated with vehicle control or Ft-3 (100 µg/mL) or F-6 (50 µg/mL) for 2.5 h. (**A**) Levels of phosphorylation of 10 proteins were detected in the lysates of these cells using Luminex assay (*n* = 2). (**B**) Data are also presented as a heat map normalized with vehicle controls and presented as fold differences. (**C**,**D**) Total protein levels of 11 proteins were detected in the lysates of these cells using Luminex assay (*n* = 3–6). (C) As of A, but for total proteins. (D) As of B, but for the fold differences in total protein levels.

Analyses of phosphoproteins in MDA MB-231 breast cancer cell line indicated that Ft-3 exerted a similar phosphoprotein response. Ft-3 increased the phosphorylation of MEK1, Erk, p38, JNK, MSK1, but suppressed p53 and c-Jun (Figure 3A,B), suggesting that Ft-3 suppresses cell growth by suppressing AP-1 protein signaling.

Total protein analyses showed that Ft-3 suppressed many components that promote cell survival as well as the components of PI3K-Akt-mTOR pathway, and Akt-mediated prevention of apoptosis (Figure 3C,D).Reduction in any of these proteins could lead to the suppression of multiple pathways to reduce cell proliferation, and to induce apoptosis. Since Ft-3 contains multiple lipids, different components could regulate specific molecular pathway(s) to induce Ft-3-mediated death in MDA MB-231 cells.

By contrast, F-6 significantly increased the activation of all three MAPKs (ERK, p38, and JNK), and MSK1 (Figure 3A,B). Consistent with JNK activation, its target c-Jun also showed increased phosphorylation. Since Erk and MSK1 were activated, c-Fos phosphorylation and AP-1 assembly would occur. However, analyses of total protein levels showed that F-6 significantly suppressed almost all the components of PI3K-Akt-mTOR pathway (Figure 3C,D). Reduction in Akt would lead to the increase in the availability of BAX to promote mitochondrial membrane leak and subsequent cell death by intrinsic pathway of apoptosis. Hence, F-6 primarily induces apoptotic cell death via Akt:BAX-mediated pathway in MDA MB-231 cell line. 

### 3.5. Apoptosis

**Figure 4:**
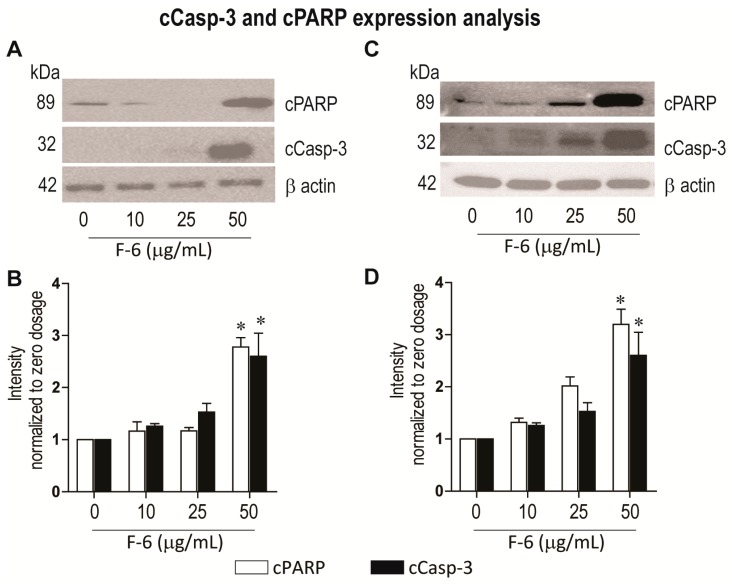
Western blot analyses show that F-6 dose-dependently induces the activation of apoptosis in leukemic K562 (**A**–**B**) and breast cancer MDA MB-231 (**C**–**D**) cells, as examined by increases in cleaved PARP and cleaved caspase 3. (**A**) Same amount of proteins from leukemic K562 cultures were lysed, size-fractionated and probed with respective antibodies. (**B**) The protein bands on the blots were quantified and standardized to beta-actin loading control. *, *p* < 0.05 compared to control. (**C**) Same amount of proteins from breast cancer MDA MB-231 cell cultures were lysed, size-fractionated and probed with respective antibodies. (**D**) The protein bands on the blots were quantified and standardized to beta-actin loading control. *, *p* < 0.05 compared to control.

To directly determine whether F-6 induces apoptotic cell death in these cell lines, we analyzed two key proteins that are hallmarks of apoptosis [31]. Activation of apoptosis is expected to induce the cleavage of the executioner caspase 3 (cCasp-3). This cCasp-3 cleaves and activates or disables various proteins including PARP. Western blot analyses showed that F-6 dose dependently induced the cleavage of both of these proteins in K562 leukemic and MDA MB-231 breast cancer cell lines (Figure 4). The highest activation of caspase 3 and degradation of PARP were detected at the F-6 concentrations used in the cell death assays (50 μg/mL; Figure 1; Figure 4). These studies confirmed that F-6 induced apoptosis in both leukemic and breast cancer cell lines.

### 3.6. Cell Recovery

**Figure 5:**
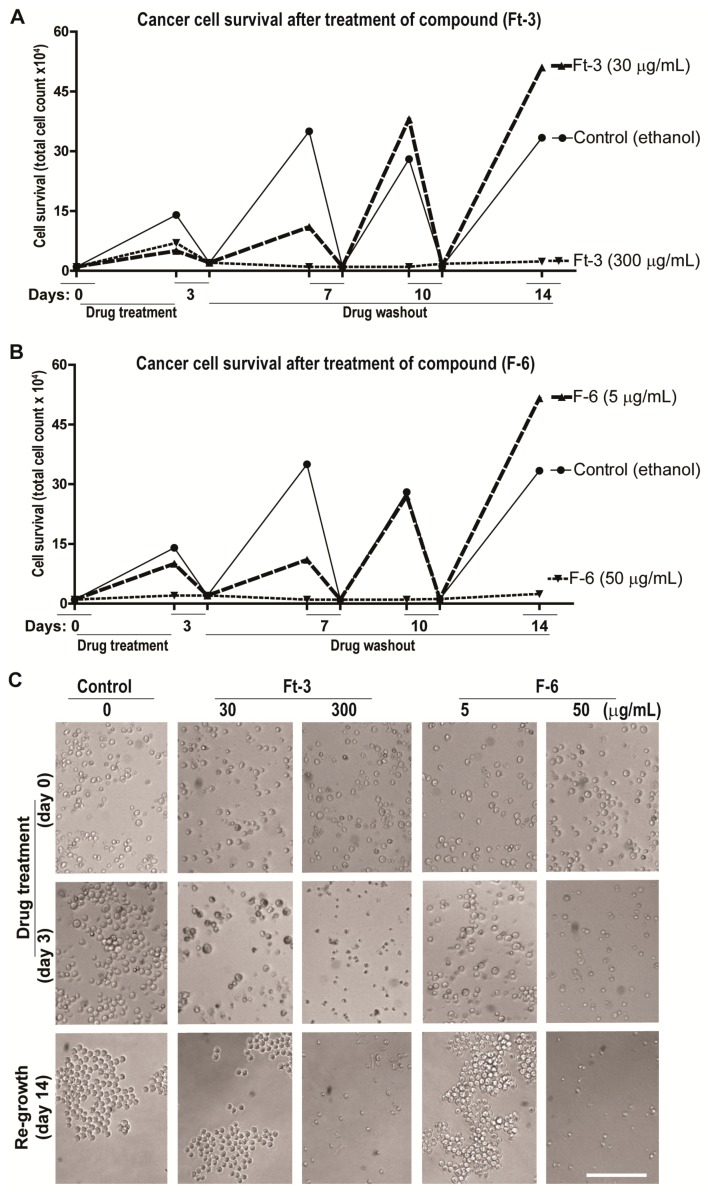
F-6 dose dependently inhibit the cancer cell recovery. Recovery of K562 cells after treatment with test compounds for 3 days and further incubation for 14 days (successive 3 days washout) in the absence of the compounds to investigate cell recovery. Line diagrams of the effect of 2 doses of Ft-3 (panel **A**) and F-6 (panel **B**) on cell numbers at the end of the first 3-day treatment of the cells with the compounds, and after each of 3 successive days in the absence (washout) of the cells after reducing the number of cells in the control to those of the treated cells. Panel **C** shows light microscopy images (× 20 magnification) of the cells at the end of several of the 3 time points. See supplemental Figure S3 for images from all time points at two concentrations of the test compounds.

Cancer cells have the ability to recover from apoptosis and proliferate. Therefore, to determine whether cancer cells have the ability to overcome Ft-3- and F6-mediated induction of apoptosis, we used K562 cells. In a set of experiments, we incubated K562 cells separately with different concentrations of Ft-3 and F-6 for 3 days along with the vehicle control, after which the medium was replaced in the absence of the compounds. This procedure of medium replacement was carried out 3 times with media changes every 3 days (14 days). At each point, we measured the cell number microscopically to observe the amount of live versus dead cells in each condition. Importantly we reduced the number of cells in the control (vehicle)-treated cells to that of the compound-treated wells after each 3-day period to measure residual activity of the compounds in the treated cells and control cells for reduced cell number and subsequent new cell growth (recovery). The reason for showing the behavior of the control cells to reduction in cell number is to indicate that untreated cells behave undamaged as expected. Ft-3 and F-6 at a dose of 300 μg/mL and 50 μg/mL, respectively completely prevented the cells from recovering during 4 washout periods of 3 days each. In contrast at 1/10th the dose, cells began to recover with F-6 and Ft-3 (Figure 5 and supplement Figure S3). Therefore, depending on the dose used, F-6 prevented cancer cell recovery from apoptosis, suggesting that F-6 is a potent anti-cancer drug candidate. 

### 3.7. Tailfin Regeneration

To see the effect of compounds (Ft-3 and F6) in vivo, the model of tailfin dissection and regeneration has been used. The tailfin was dissected, and regeneration of the tailfin was followed by measuring the tailfin length at 2 and 6 days post amputation in presence and absence of compounds (Ft-3, F6, and vehicle control). The medium and the compounds were changed every two days. The images and measurement data represented in Supplementary Figure 4 show the effect of F6 on tailfin regeneration. Importantly this shows that the compound is not toxic at the doses used. 

## 4. Discussion

**Figure 6:**
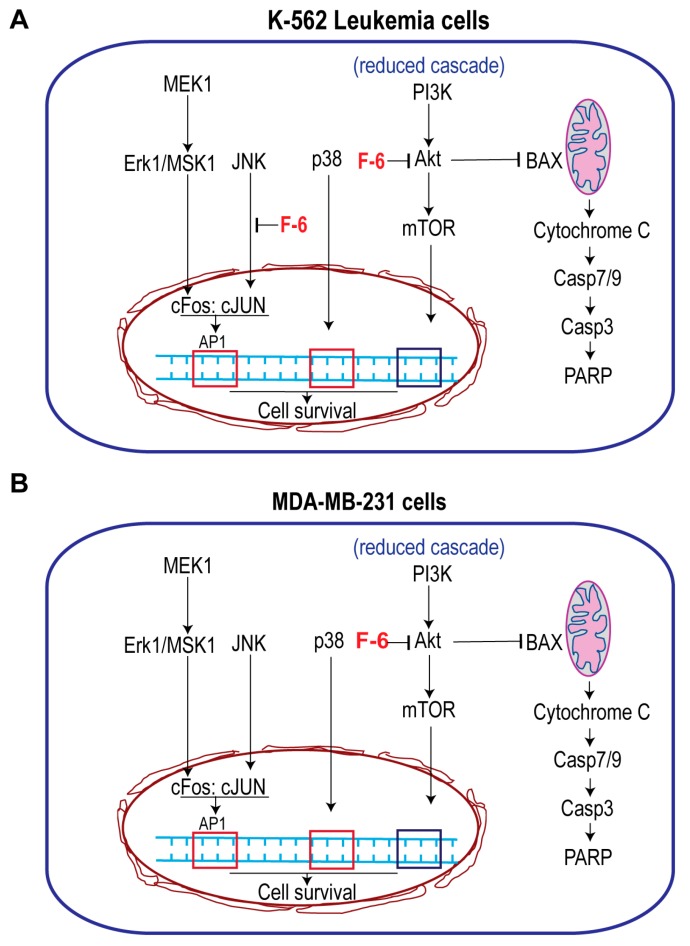
Simplified pathways showing the effect of F-6 cell proliferation and death. (**A**) Leukemic cell line K562 cells. F-6 suppresses cell proliferation by inhibiting JNK activity that prevents c-Jun activation, and PI3K-Akt-mTOR pathway. It also suppresses Akt:BAX-mediated mitochondrial protection and promotes intrinsic pathway of apoptosis. (**B**) MDA MB-231 breast cancer cells. F-6 suppresses cell proliferation by inhibiting PI3K-Akt-mTOR pathway. It also suppresses Akt:BAX-mediated mitochondrial protection and promotes intrinsic pathway of apoptosis. Therefore, F6 suppresses cell proliferation and promotes apoptotic cell death in both of these types of cancer cells.

Preparation from the epidermal gel secretions of the Arabian Gulf catfish (*Arius bilineatus Val.*; AGCS) has many therapeutically bioactive compounds. However, the anti-cancer property of this material is unknown. We report in this study that a bioactive lipid component, F-6, of the epidermal gel secretions of AGCS has potent anti-cancer properties. Results presented herein indicate that the lipid fraction of AGCS (Ft-3) and a component F-6 of Ft-3 have anti-cancer activities on three human cancer cell lines, in vitro. Our studies indicate that both Ft-3 and F-6 had anti-proliferative and pro-apoptotic activities (Figure 1). These compounds, particularly F-6, suppresses cell proliferation by inhibiting JNK-mediated pathways, inducing apoptosis by suppressing Akt mediated mitochondrial protection (Figure 2 and Figure 3). These pathways culminate in the induction of caspase 3-mediated intrinsic pathway of apoptosis (Figure 4). Both Ft-3 and F-6 prevent recovery of cancer cells in a dose-dependent manner (Figure 5). Collectively, the dose response data of compound suggests apoptosis or anti-proliferative action; however, the multiplex kinase and expression data clearly show the hallmarks of apoptosis (cPARP and cCasp-3) an effect which is not reversible at the higher doses. Therefore, F-6 is a novel lipid molecule that has anti-cancer properties with anti-proliferative, pro-apoptotic and recovery-preventing abilities (Figure 6). The proposed pathways are suggested based on published information, our Luminex data and expression analyses of cCasp-3 and cPARP proteins. 

Fish and mammalian cells have many similar properties. However, certain cytotoxic molecules have drastically different cytotoxic properties between these two cell types (10–100-fold difference; e.g., paraquat, potassium chloride, xylene, and dichloromethane [32]. Therefore, some of the molecules commonly present in the fish dermal preparations could have a potent cytotoxic effect on mammalian cancer cells. Our studies show that the lipid extract of AGCS had a dose-dependent cytotoxic effect on three different human cancer cell lines (Figure 1). F-6 is a minor component of Ft-3, but had a drastic pro-NETotic activity [9]. In the current study, F-6 shows 3.5 and 16-fold more effective killing of breast cancer cells and leukemic cells, respectively, than Ft-3 (Figure 1), suggesting that F-6 is a major active ingredient of the Ft-3. 

Anti-cancer compounds could suppress cell proliferation, induce cell death, or exert both of these effects [33]. Our multiplex analyses of protein phosphorylation and changes in the expression of key cell-death related proteins show that Ft-3 and F-6 possess both anti-proliferative and pro-death activities. F-6 induced the activation of all three MAP kinases (Figure 2 and Figure 3). F-6 also activated Erk and JNK during NET formation because the inhibitors of these kinases suppressed F-6-mediated NETosis [9]. JNK is a key MAPK that phosphorylates c-Jun, and subsequently activates AP-1-induced proliferative gene expression in cancer cells [34]. F-6 specifically suppresses JNK-mediated c-Jun activation in K562 leukemic cells but not in MDA MB-231 breast cancer cells (Figure 2 and Figure 3), hence, this lipid could suppress cell proliferation in a cancer-cell type specific manner. PI3K-Akt-mTOR is another major cell proliferative pathway [35]. Nevertheless, multiplex assay data indicate that F-6 suppresses the expression of several proteins that are involved in the activation of PI3K-Akt-mTOR pathway (Figure 2 and Figure 3). Therefore, F-6 suppresses PI3K-Akt-mTOR pathway to suppress cell proliferation in both of these cell lines. Ft-3 shows substantially different effects on cell proliferation pathways, suggesting that it has other components that may regulate cell proliferation by different pathways.

Anti-cancer compounds could kill cells by inducing various forms of death pathways [36]. Akt, of the PI3K-Akt-mTOR cascade, is a major regulator of apoptotic cell death [35]. Akt binds and sequesters BAX, a mitochondrial membrane-permeability increasing protein, to prevent apoptosis [37]. F-6 suppressing Akt levels indicate that F-6 could induce apoptosis (Figure 2 and Figure 3). By inducing the activation of caspase 3 (cCasp-3) in both leukemic and breast cancer cell lines confirms that F-6 is truly pro-apoptotic (Figure 4).

Ft-3 shows the activation of slightly different pathways suggesting that other lipid components present in the mixture may have additional compounds with cytotoxic properties. Overall, lytic forms of cell death is inflammatory and often not preferred for killing cancer cells [38]. By contrast, apoptotic cell death is anti-inflammatory and apoptotic cells are readily cleared by adjacent cells or phagocytes [38]. Therefore, F-6 could be a viable anti-cancer molecule.

Studies conducted in the last decade indicate that cancer cells that undergo apoptotic cell death could revert its death program and regrow in a process termed as anastasis [19,20]. Our studies show that such recovery could be prevented by F-6 in a dose dependent manner (Figure 5, Figure S1). As expected, Ft-3 was less efficient compared to pure F-6 in preventing cancer cell recovery. Importantly this compound is not toxic to the zebrafish tested during the tailfin regeneration after 2 and 6 days post-amputation. Therefore, F-6 represents an anti-cancer molecule with a good therapeutic potential.

F-6 belongs to a family of fatty acids containing a furan ring in the middle of the fatty chain. They are found in algae, but also in plants and certain microorganisms [8,10,11,39,40,41,42,43]. They are present in triglycerides and phospholipids as esters [44], and are also detected in human urine [45]. Furan fatty acids are believed to serve a role as antioxidants as they act as radical scavengers [46]. They are highly reactive, forming thioethers with glutathione and cysteine [47]. The biological roles of these special fatty acids formed from polyunsaturated fatty acids is not known although an effect on MCF-7 cancer cells in vitro has been reported from the viewpoint of estrus [48]. Synthetic chemistry of F-6 has been reported [49]. Therefore, it may be possible to modify F-6 and related furan-containing fatty acids to increase their potency for specific disease indications.

## 5. Conclusions

We describe for the first time that F-6, one component of the lipid fraction of AGCS (Ft-3), potently suppresses cell proliferation of various human cancer cells in vitro, induces apoptosis and prevents recovery after the treatment. This report further describes that F-6 (12,15-epoxy-13,14-dimethyleicosa-12,14-dienoic acid), prevents cancer cell proliferation (e.g., blocking JNK pathway), induces apoptotic cell death (e.g., suppressing Akt pathway) and prevents cell recovery, in a dose-dependent manner. Therefore, this study uncovers F-6 as a potential anti-cancer drug candidate. In view of the previously described safety of the total epidermal AGCS preparation in man (anti-inflammatory and wound healing actions) [7,8], F-6 may find use in the treatment of several forms of cancer. These results further demonstrate that F-6, at least in part, contributes to the therapeutic activities of preparations from the total epidermal secretion isolated from the catfish.

## Supplementary Materials

The following are available online at https://www.mdpi.com/2072-6694/11/7/960/s1, Table S1: Regression equations, r^2^ and *p*-values for Ft-3 and F-6 mediated cell death parameter for K562 and MDA MB-231 cells, Figure S1: Data taken from Figure 1 and linear regression analyses performed, Figure S2: Ft-3 and F-6 dose dependently inhibit the cancer cell recovery, Figure S3: A single cut is made traversing the entire dorsoventral length of the caudal fin through the end of the notochord.

## Abbreviations

MAPKs	Mitogen-Activated Protein Kinases
Erk	Extracellular Signal-Regulated Kinase
JNK	Jun N-Terminal Kinases
PI3K/Akt/mTOR	Phosphatidylinositol-3-Kinase/Protein Kinase B/mammalian Target of Rapamycin
ADP	Adenosine Diphosphate
