# Supplementary material for: Furanoic Lipid F-6, A Novel Anti-Cancer Compound that Kills Cancer Cells by Suppressing Proliferation and Inducing Apoptosis
Source: Cancers (Basel). 2019 Jul 9;11(7):960. doi: 10.3390/cancers11070960 (PMC6678287; doi:10.3390/cancers11070960)
Supplement: Supplementary file 1 [file cancers-11-00960-s001.pdf]

Supplementary Materials: Furanoic Lipid F-6, A Novel Anti-Cancer Compound that Kills Cancer Cells by Suppressing Proliferation and Inducing Apoptosis

Jassim M. Al-Hassan, Yuan Fang Liu, Meraj A. Khan, Peiying Yang, Rui Guan, Xiao-Yan Wen, Mohammad Afzal, Sosamma Oommen, Bincy M. Paul, Divya Nair, Nades Palaniyar and Cecil Pace-Asciak

Table S1. Regression equations, r^2 and *p*-values for Ft-3 and F-6 mediated cell death parameter for K562 and MDA MB-231 cells.

Data	Cell types	Treatments (µg/mL)	Slope %/[µg/mL]	Y-intercept %	r^2	*p*-Value
**Cell death**	K-562	Ft-3 (100)	0.79	7.87	0.42	0.0080
		F-6 (50)	18.90	2.41	0.96	0.0001
	MDA-MB231	Ft-3 (100)	8.42	1.75	0.85	0.0001
		F-6 (50)	18.23	7.21	0.97	0.0001
	MCF-7	Ft-3 (100)	3.01	26.37	0.32	0.0022
		F-6 (50)	11.02	29.29	0.53	0.0001
**Cell Survival (colorimetric)**	K-562	Ft-3 (100)	−1.86	94.93	0.25	0.0080
		F-6 (50)	−15.07	94.18	0.69	0.0340
	MDA-MB231	Ft-3 (100)	−3.13	97.43	0.34	0.0104
		F-6 (50)	−15.60	91.66	0.75	0.0001
	MCF-7	Ft-3 (100)	−5.27	101.12	0.68	0.0001
		F-6 (50)	−16.70	96.31	0.85	0.0001

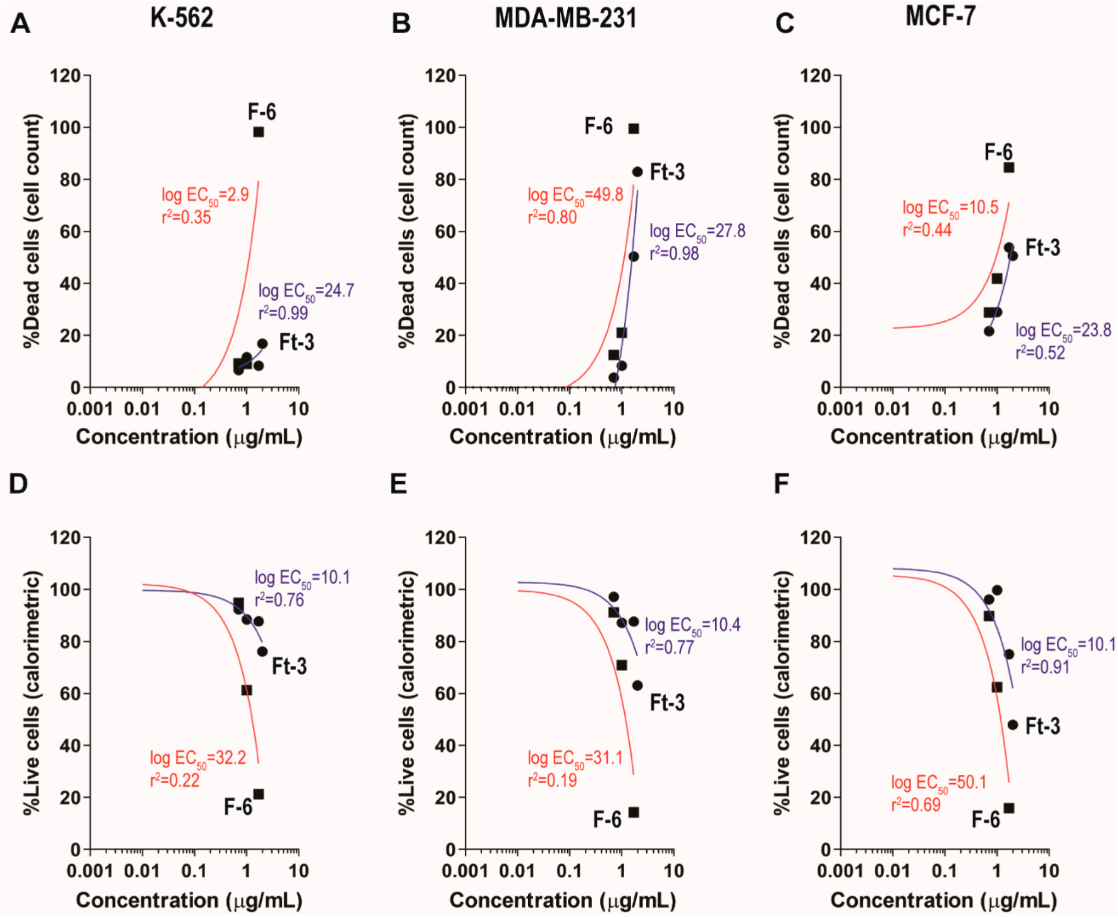

Figure S1. Taken from Figure 1 and linear regression analyses performed. (**A**–**C**) Nonlinear regression (curve fit) with the equation of log10 (drug) vs response were applied to calculate EC50 values in each condition. Analyses show that both Ft-3 and F-6 dose-dependently kill all three cancer cells. The r^2 values and logEC50 values of compounds in each condition are indicated on each panel. Data show that both Ft-3 and F-6 dose-dependently kill all three cancer cells. (**D**–**F**) Anti-proliferative activity of Ft-3 and F-6 was also analyzed with the WST-1 spectrophotometric detection method (n = 3).

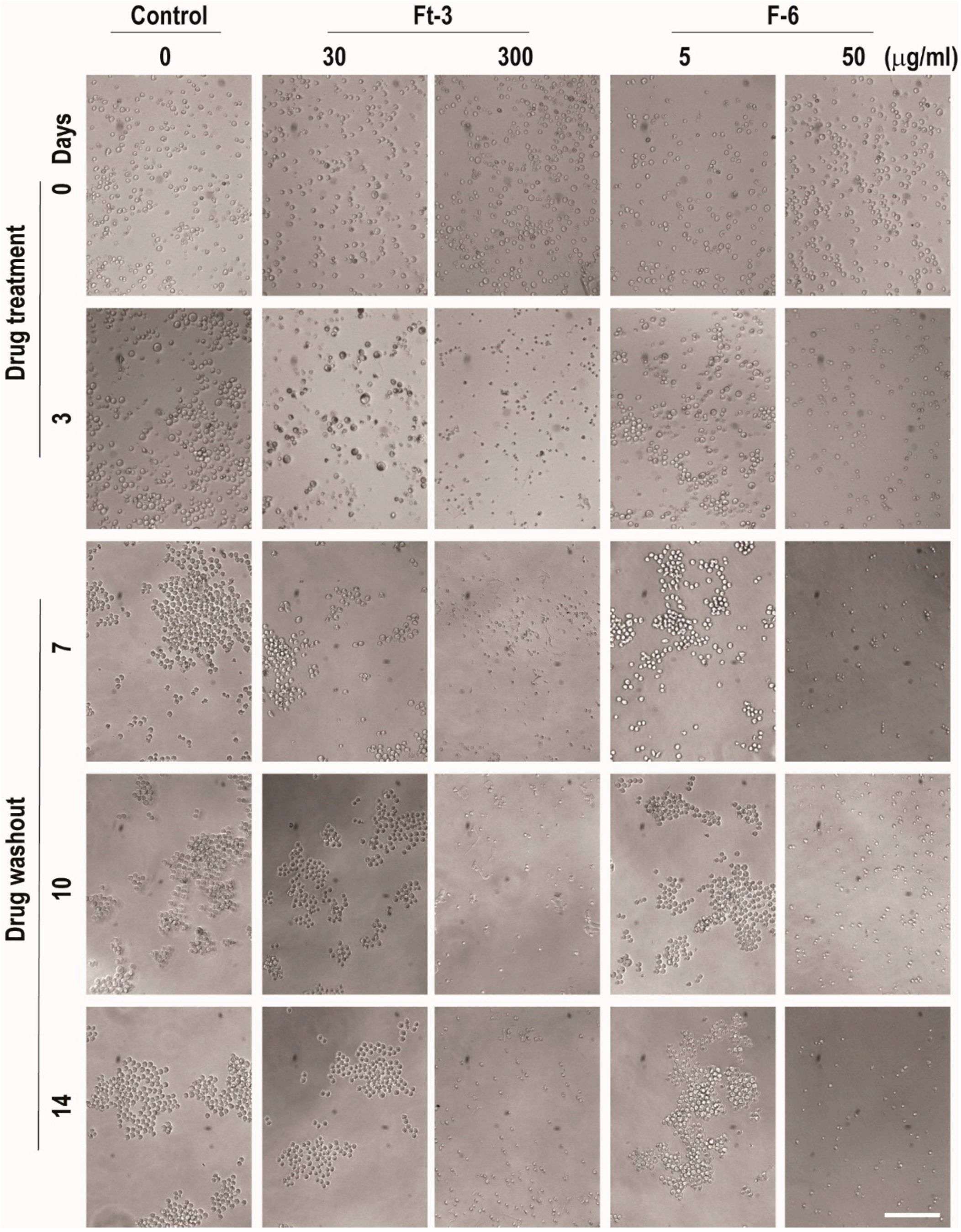

Figure S2. Ft-3 and F-6 dose dependently inhibit the cancer cell recovery. Microscope images (× 20 magnification) of K562 cells at two doses indicated of Ft-3 and F-6 used and at all time points. Shown are images of the cells before the start of treatment (day 0, 1e4 cells), at the end of the 3 day treatment (day 3, variable cell numbers depending on treatment) and subsequent days indicated of drug washout before cells were diluted to those wells showing the least cell number. Results show Ft-3 and F-6 dose-dependent inhibition of cell recovery.

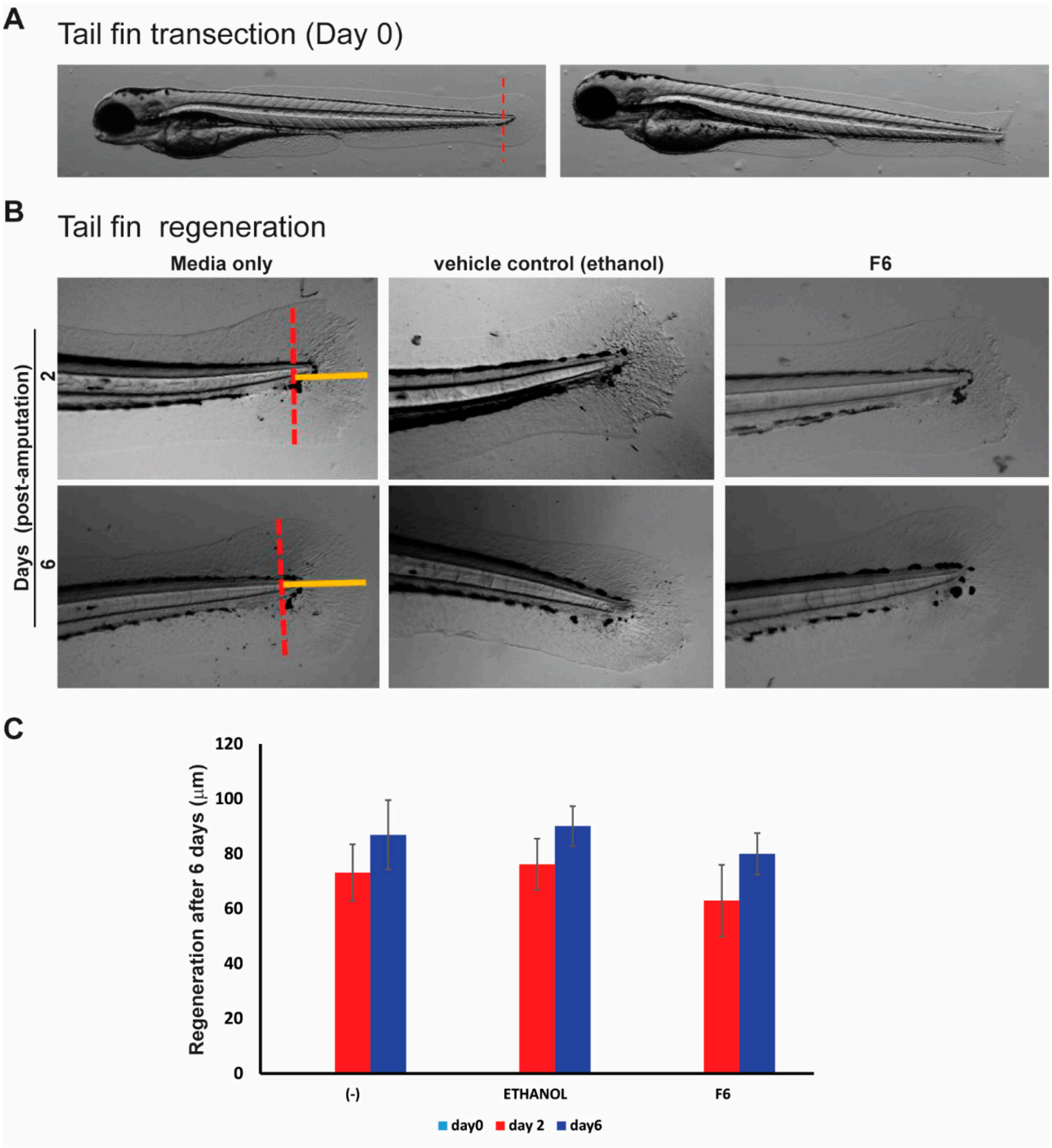

Figure S3. A single cut is made traversing the entire dorsoventral length of the caudal fin through the end of the notochord. Fish larvae with tailfin uncut are maintained in 24-well plastic dishes with only media, vehicle control (ethanol) and F6 for 2 and 6 days at 28°C. Images are acquired using fluorescence stereomicroscopy (Leica M205 FA). The lengths of tailfin are analyzed using Fiji software. Data show that the regeneration is not affected by F6 and importantly the compound is not toxic to the fish.
